# Ecological niche differentiation mediates near complete premating reproductive isolation within the *Gladiolus carneus* (Iridaceae) species complex

**DOI:** 10.1093/aob/mcaf172

**Published:** 2025-07-24

**Authors:** Katharine L Khoury, Shelley Edwards, Ethan Newman

**Affiliations:** Department of Botany, Rhodes University, Makhanda 6139, South Africa; Department of Zoology and Entomology, Rhodes University, Makhanda 6139, South Africa; Department of Botany, Rhodes University, Makhanda 6139, South Africa

**Keywords:** Niche differentiation, premating reproductive isolation, ecogeographic isolation, pollinator-mediated isolation, phenological isolation, Cape Floristic Region

## Abstract

**Background and Aims:**

Ecological niche differentiation is well associated with intraspecific divergence of functional traits, which may lead to the evolution of premating reproductive isolation. However, the link between the ecological niches, trait divergence and premating isolation remains poorly understood. This is particularly pertinent in hyperdiverse areas, such as the Cape Floristic Region of South Africa, where fine-scale ecological heterogeneity has been hypothesized as a major driver of speciation. Using the polymorphic geophyte *Gladiolus carneus*, endemic to the Cape Floristic Region, we test whether ecological niche differentiation mediates premating reproductive isolation.

**Methods:**

We first tested whether putative ecotypes of *G. carneus* were distinct based on their floral and vegetative morphology. Next, we documented the abiotic niche, flowering phenology and pollination niche of each putative ecotype and tested whether any resulting niche differentiation causes premating reproductive isolation.

**Key Results:**

Seven distinct ecotypes were identified. Using niche modelling and multivariate analyses, we found that these ecotypes occupied distinct abiotic niches, resulting in strong ecogeographic isolation. They also had distinct flowering times, causing varying strengths of phenological isolation. For the pollinator niche, we found that all sampled populations were pollinated by one of three highly effective functional pollinators; however, at the ecotypic level there were no consistent trends, leading to varying strengths in pollinator-mediated isolation. Across all ecotypes, ecogeographic isolation was the strongest barrier to gene flow, which, combined with phenological and pollinator-mediated isolation, caused near complete premating reproductive isolation.

**Conclusions:**

These results suggest that ecological niche differentiation between *G. carneus* ecotypes might be contributing to incipient speciation within the species complex and further suggest that ecological niche differentiation may be a major driver of speciation in the hyperdiverse Cape Floristic Region.

## INTRODUCTION

At the early stages of ecological speciation, ecologically based divergent selection is key in producing phenotypes that often associate with distinct biotic and abiotic niches (Schluter, 2000, 2001; Rundle and Nosil, 2005). These divergent niches occupied by distinct phenotypes are often directly associated with gene flow barriers that act before mating (Coyne and Orr, 2004; Rundle and Nosil, 2005). In seed plants, these premating barriers are thought to be important in completing reproductive isolation, especially in recently diverged taxa or ecotypes (Christie *et al.*, 2022), making it pertinent to disentangle the ecological shifts underlying barrier effectiveness when studying lineage diversification. For example, if shifts in abiotic factors cause divergent selection on the functional traits of a plant in different parts of its range, it will likely result in ecogeographic isolation, which is defined as a reduction in encounter rates owing to spatial separation as a result of genetically based differences (Sobel, 2014). Likewise, closely related taxa associated with distinct pollinator niches can have divergent floral phenotypes that promote pollinator-mediated isolation (Kay, 2006; Sobel and Streisfeld, 2015; Minnaar *et al.*, 2019). Specifically, this can occur when niche differentiation is associated with functional pollinator groups that differ in their sensory systems, causing pollinator-mediated isolation through attraction traits (e.g. flower colour) (Grant, 1994; Lunau *et al.*, 2011; Newman *et al.*, 2012). Alternatively, differences in pollinator dimensions can cause pollinator-mediated isolation through morphological-fit floral traits (e.g. corolla tube length), which can be accompanied with or without differential pollen placement (Minnaar *et al.*, 2019; Newman and Anderson, 2020). Here, a reduction in gene flow is linked directly to differences in pollinator functional groups and how they interact with the floral traits of closely related taxa, making it pertinent to study both the pollinator niche and their functional fit with floral traits. Phenotypic shifts, such as flowering times, are difficult to associate with any single biotic or abiotic driver. Shifts in flowering phenology can be attributed either to selection by biotic and abiotic factors (Elzinga *et al.*, 2007; Munguia-Rosas *et al.*, 2011) or to plastic responses within the abiotic environment (Elzinga *et al.*, 2007). Regardless of the causes of the shift in flowering times, it can result in phenological isolation (Paudel *et al.*, 2018; Ramirez-Aguirre *et al.*, 2019), which is a reduction in gene flow attributable to a mismatch in flowering times (Coyne and Orr, 2004).

Although ecological shifts in association with early lineage diversification have been well documented (Grossenbacher *et al.*, 2014; Dellinger *et al.*, 2024; Fernandez-Mazuecos and Glover, 2025), this is rarely placed in the context of reproductive isolation (but see Sandstedt *et al.*, 2021; Boucher *et al.*, 2023; Farnitano and Sweigart, 2023). Usually, reproductive isolation is studied either by quantifying the strength of multiple barriers between sister taxa (see Ramsey *et al.*, 2003; Kay, 2006; Sobel and Streisfeld, 2015; Ostevik *et al.*, 2016; Christie and Strauss, 2019; Ivey *et al.*, 2023) or by focusing on a single barrier across multiple pairs of taxa (Sobel, 2014). The first approach provides valuable information on the relative strength of barriers between pairs of taxa, whereas the second approach shows the role of single barriers in driving wider trends in reproductive isolation, both within and between lineages. If these approaches were to be combined, with multiple gene flow barriers quantified to test their relative importance across multiple taxa pairs, it would show which barriers played the largest role in maintaining reproductive isolation and driving the diversification of lineages as a whole. This approach has highlighted the role of multiple postpollination barriers (particularly hybrid seed inviability) in reducing gene flow between *Mimulus* species in the section *Eunanus* (Farnitano and Sweigart, 2023), for postzygotic barriers completing reproductive isolation in the *Mimulus tilingii* species complex (Sandstedt *et al.*, 2021) and for showing that geographic and phenological isolation maintains species boundaries within the genus *Argyroderma* (Boucher *et al.*, 2023).

Documenting ecological niche differentiation in association with premating isolation is particularly pertinent in hyperdiverse areas, such as the Cape Floristic Region (CFR), where interactions between topography, soils, climate and pollinators have generated ecological heterogeneity that has facilitated the diversification of plant lineages (Ellis *et al.*, 2014). Specifically, the CFR is characterized by folded mountain belts that run parallel to the Indian and Atlantic ocean coasts (Linder, 2003), which gave rise to some of the most nutrient-poor soils currently recorded on Earth (Cramer *et al.*, 2014). However, these soils contrast with the moderately fertile, shale-derived soils in coastal planes and intermontane valleys (Cramer *et al.*, 2014), which have created a mosaic of edaphic conditions in the CFR. Rainfall varies along both an east–west gradient and an elevational gradient. In the west, there is a strongly seasonal mediterranean climate, whereas in the east, the climate is much more aseasonal, with rainfall throughout the year (Manning and Goldblatt, 2012). Further variation is created by steep, mountainous landscapes, resulting in orographic rainfall (Manning and Goldblatt, 2012). These interacting conditions have created a heterogeneous abiotic selective landscape that has facilitated frequent ecological speciation in the CFR (van der Niet and Johnson, 2009; Schnitzler *et al.*, 2011; Ellis *et al.*, 2014). Apart from diverse abiotic niches that have contributed to lineage diversification, shifts in biotic niches associated with pollinators are also thought to be important drivers of lineage diversification in the Cape (van der Niet and Johnson, 2009; Valente *et al.*, 2012). These ecological shifts considering both biotic and abiotic niches have been shown at a macroevolutionary scale, focusing on species-level phylogenies of the major Cape clades (van der Niet and Johnson, 2009; Schnitzler *et al.*, 2011; Valente *et al.*, 2012), and through trait-by-environment associations (Carlson *et al.*, 2011; Mitchell *et al.*, 2015; Newman *et al.*, 2015). However, few of these ecological shifts have been linked directly to the strength of corresponding premating isolation barriers in the CFR (but see Newman and Johnson, 2025) to elucidate the relative importance of individual gene flow barriers to lineage diversification.

**Fig. 1.**
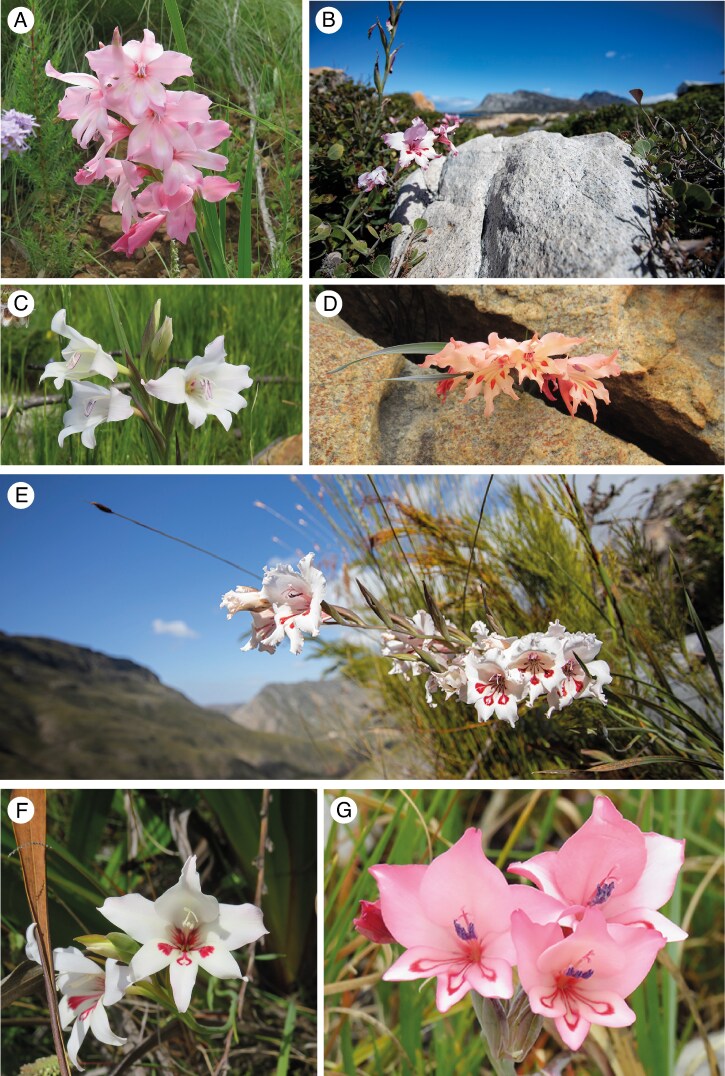
Colour plate of all *Gladiolus carneus* ecotypes. These include *albidus* (A), *blandus* (B), *callistus* (C), *high-altitude* (D), *langeberg* (E), *macowanianus* (F) and *prismatosiphon* (G). Photographs: A, C, D, F, G: Katharine Khoury; B, E: Ethan Newman.

*Gladiolus carneus* Delaroche (Iridaceae) is a geophyte endemic to the CFR (Fig. 1). The species complex is polymorphic and is made up of at least seven distinct forms that differ in both their functional traits and geographic distributions (Lewis *et al.*, 1972; Delpierre and du Plessis, 1974), representing putative ecotypes (Turesson, 1922). This provides an opportunity to explore the relationship between trait divergence, ecological niche differentiation and premating reproductive isolation in a diverging species complex within the CFR. Using the *G. carneus* study system, we tested: (1) whether putative ecotypes are morphologically distinct from one another; (2) whether they occupy distinct abiotic, phenological and pollinator niches; and (3) whether differences in these ecological niches result in premating reproductive isolation.

## MATERIALS AND METHODS

### Study species

*Gladiolus carneus* is a deciduous cormous geophyte endemic to the CFR. The species occurs from Ceres, south into the Cape peninsula, and east along the Southern Cape coast and then inland into the Langeberg and Outeniqua Mountains (Goldblatt and Manning, 1998). *Gladiolus carneus* occurs along the entire elevational gradient of the CFR, with populations occurring from sea level along the rocky Kleinmond coast, up to the high mountain peaks of the South-Western and Southern Cape, >1000 m above sea level. The species flowers from August into early January (Lewis *et al.*, 1972). *Philoliche rostrata* (Tabanidae) and *Prosoeca nitidula* (Nemestrinidae), both long-tongued flies, have been documented pollinating the species (Goldblatt and Manning, 1999; Goldblatt *et al.*, 2001), whereas *Amegilla spilostoma* (Apidae), a solitary bee, is a documented nectar thief (Goldblatt *et al.*, 2001). However, these observations were conducted at only two sites (Devil's Peak and Silvermine), and our own preliminary observations suggest that both long-tongued flies and solitary bees are legitimate pollinators of *G. carneus*.

*Gladiolus carneus* has been described under ≥20 different names, with many of those taxonomic distinctions referring to specific populations or forms of the species. These forms were eventually sunk into the single species, *Gladiolus carneus*, by Lewis *et al.* (1972) . However, these forms are likely to represent ‘putative ecotypes’ based on their distinctive morphology and geographical distributions, and we provide empirical evidence for ecotype status in the manuscript. Specifically, in the Materials and Methods, we refer to the different forms as ‘putative ecotypes’ and provide evidence in the Results. The results of this study provide evidence for seven ecotypes, five of which have been included previously by Delpierre and du Plessis (1974) as varieties, and we provide evidence for a further two. The putative ecotypes include *albidus*, *blandus*, *callistus*, *macowanianus* and *prismatosiphon* as defined by morphology and localities described by Delpierre and du Plessis (1974). The form of *G. carneus* that has been found in the Langeberg mountains has been grouped previously in both *blandus* (Delpierre and du Plessis, 1974) and *prismatosiphon* (Lewis *et al.*, 1972). However, owing to its distinct geographical range and floral morphology (this study), the Langeberg form of *G. carneus* has been treated as a separate putative ecotype. Likewise, the late-flowering, high-elevation forms of *G. carneus* in the Drakenstein, Hottentots-Holland and Riviersonderend mountain ranges were treated as a *high-altitude* putative ecotype.

*Albidus* (Fig. 1A) occurs in Paarl, Stellenbosch and south into Hermanus (Delpierre and du Plessis, 1974). The ecotype can be identified by the white flowers with pale yellow markings on the lower tepals (Delpierre and du Plessis, 1974). *Blandus* (Fig. 1B) is found near Kleinmond (Delpierre and du Plessis, 1974). *Callistus* (Fig. 1C) occurs widespread across the Cape and lacks any nectar guides, and instead has a deep purple gullet (Delpierre and du Plessis, 1974). The *high-altitude* ecotype includes all late-flowering populations occurring on the top of the Drakenstein, Hottentots-Holland and Riviersonderend mountain ranges (Fig. 1D), which are frequently exposed to summer cloud cover and freezing temperatures in winter. The *langeberg* ecotype of *G. carneus* is found in the Langeberg and Outeniqua mountains (Fig. 1E). *Macowanianus* is the most well-documented ecotype (Fig. 1F), found in the Southern Cape and along the Cape peninsula (Delpierre and du Plessis, 1974), and is identified by its distinct dark nectar guides. *Prismatosiphon* (Fig. 1G) is restricted to the Agulhas Plain and is found predominantly on rocky outcrops (Delpierre and du Plessis, 1974).

### Are the putative ecotypes of *G. carneus* morphologically distinct?

#### Morphological differences between putative ecotypes of *G. carneus*

To test whether *G. carneus* separates into distinct ecotypes based on morphology, we measured morphological traits in a total of 29 populations (eight *albidus*, one *blandus*, three *callistus*, three *high-altitude*, three *langeberg*, eight *macowanianus* and three *prismatosiphon*; for site coordinates, see Supplementary Data Table S1) representing the entirety of the geographical and morphological variation within the species complex (for sample sizes, see Supplementary Data Table S2). Voucher specimens were collected from 14 populations representing six of the seven putative ecotypes, which were deposited at the Selmar Schonland Herbarium (GRA) (Supplementary Data Table S1). At each population, we chose between 5 and 40 individuals during their peak flowering season for morphological measurements. To avoid pseudoreplication, all floral and vegetative measurements were taken from a single flower and leaf per individual. Some or all of the following morphological traits were measured on each individual using digital callipers (0–200 mm, TA): floral tube length, flower gape, petal size, flower width, and width of the longest leaf; and the inflorescence height and the length of the longest leaf were measured using a measuring tape (Supplementary Data Fig. S1A–C). Flower gape was measured from the lowest anther to the lower median tepal directly underneath (Supplementary Data Fig. S1A). Flower width was the distance between the tips of the lateral tepals (Supplementary Data Fig. S1A). Floral tube length was measured from the top of the ovary to the notch connecting the corolla to the lower tepals (Supplementary Data Fig. S1B). Petal size was measured from the tip to the base of the dorsal tepal (Supplementary Data Fig. S1B). Inflorescence height was measured from the base of the inflorescence to the top of the display (Supplementary Data Fig. S1C). Leaf length was measured as the distance from the ground to the tip of the longest leaf (Supplementary Data Fig. S1C), and leaf width was measured as the widest point on the longest leaf (Supplementary Data Fig. S1C). Finally, we documented the total number of flowers, which included all buds, open and senesced flowers, and the total number of leaves starting from the base of the fan during the peak flowering period.

All data analysis was conducted in R v.4.1.2 (R Core Team, 2021). We used a series of principal component analyses (PCAs) to determine whether different putative ecotypes cluster separately based on their vegetative and floral morphology. We implemented the PCAs using the *prcomp* function from the ‘*stats*’ package (R Core Team, 2021) and tested whether the putative ecotypes cluster separately using a permutation MANOVA (999 permutations). We then used a pairwise permutation MANOVA with a Bonferroni correction for pairwise comparisons between putative ecotypes. The permutation MANOVA was implemented using the *adonis2* function in the package ‘*vegan*’ (Oksanen *et al.*, 2020), and we used *pairwise.perm.manova* from the ‘*RVAideMemoire*’ package (Herve, 2023) for the pairwise comparisons. We used this procedure to test whether the putative ecotypes separate based on their floral traits, vegetative traits and all morphological traits.

We also determined whether there were significant differences between putative ecotypes using generalized linear models (GLMs) implemented in the ‘*stats*’ package (R Core Team, 2021) for the following morphological traits: tube length, flower gape, petal size, inflorescence height, and length and width of the longest leaf. These traits were chosen because they represent functional traits directly linked to the biological variation associated with their biotic and abiotic niches (e.g. tube length is associated with pollinator proboscis length). In the models, each trait was set as the response variable, while the explanatory variables were set as ecotype, and site nested within ecotype. The nested term was included to account for sampling at multiple sites per ecotype. All traits were fitted with either a Gaussian or gamma error distribution, depending on the normality of the data set. Significances of model terms were obtained using the *Anova* function from the ‘*car*’ package (Fox and Weisberg, 2019), whereas the *emmeans* function from the ‘*emmeans*’ package (Lenth, 2022) was used for pairwise comparisons.

#### Colour differences between putative *G. carneus* ecotypes

To test whether there were differences in the colour traits between putative ecotypes of *G. carneus* within bee and fly colour vision, we sampled 1266 spectral measurements from 300 individuals across 24 populations in 2020, 2022 and 2023. Within each population, we took spectral measurements from between 3 and 28 individuals (see sample sizes in Supplementary Data Table S3). All spectral data were collected within the same years as all morphological data. For each individual of the *macowanianus*, *blandus*, *prismatosiphon*, *high-altitude* and *langeberg* putative ecotypes, on a single flower we measured the spectral reflectance of the dorsal tepal, and the outline and centre of the nectar guides (hereafter referred to as the ‘guide’ and ‘centre’) on the lower median tepal and lower lateral tepal (Supplementary Data Fig. S1D). Only the dorsal tepal and centre of the nectar guides were documented for *albidus* (Supplementary Data Fig. S1E), because the putative ecotype lacks well-developed outlines around the nectar guides. Given that *callistus* lacks any nectar guides, only the dorsal tepal and the gullet for each individual were measured (Supplementary Data Fig. S1F). All spectra collected in 2020 were measured using an Ocean Optics S2000+ spectrometer with a DT-mini light source and fibre-optic probe (ultraviolet/visible 400 μm), whereas spectra collected in 2022 and 2023 were measured using an Ocean Insight FLAME Miniature spectrometer (Ostfildern, Germany) with a PX-2 Pulsed Xenon Light Source and a Premium 400 μm fibre-optic probe. The spectra of all putative ecotypes were processed, and negative values were corrected using the *procspec* function. Mislabelled spectra or spectra with incomplete information were removed. Aggregation plots showing the mean and standard error (s.e.) of the putative ecotypes for each trait were generated using the *aggplot* function to visualize the curvature of the reflectance spectra for each species (Supplementary Data Fig. S2A–F).

Colour vision models were used to determine how pollinators perceive the colours of each putative ecotype. Reflectance spectra of each putative ecotype were plotted in the colour hexagon (Chittka, 1992) for the *Apis mellifera* visual system and the colour-opponent coding vision model for flies (Troje, 1993). Bees have a conserved trichromatic visual system, with ultraviolet, blue and green photoreceptors (Briscoe and Chittka, 2001), which are represented as vertices in the colour hexagon of Chittka (1992). Bees have continuous colour discrimination, which can be quantified by calculating the Euclidean distances between loci within the colour hexagon. These Euclidean distances are a proxy for colour contrasts, with larger distances being more perceptible than smaller distances. Given that achromatic vision is suggested to be important for object detection in bees (van der Kooi and Kelber, 2022), we calculated the achromatic contrast between loci using the green photoreceptor (Giurfa *et al.*, 1996).

Fly colour vision was modelled using the colour-opponent coding (COC) model (Troje, 1993). The COC model is based on behavioural experiments with *Lucilia* blowflies that showed their colour vision was based on an opponency mechanism involving pairs of photoreceptors, namely R7p with R8p and R7y with R8y. *Lucilia* flies distinguish colour categories that depend on the relative excitation of the paired receptors. The COC model uses the relative quantum catches of the four fly photoreceptors to plot spectra into quadrants in a Cartesian plane. Loci plotted within a quadrant are too similar to be perceived, whereas loci in different quadrants are considered perceptible colour differences. However, Hannah *et al.* (2019) found that the hoverfly, *Eristalis tenax*, can discriminate between colours within the COC quadrants and, based on these experiments, Garcia *et al.* (2022) proposed four behaviourally relevant colour discrimination thresholds for *E. tenax*. Given that the spectral sensitivities of fly pollinators of *G. carneus* are currently unknown, we used the spectral sensitivities of *E. tenax*, a widespread nectar-feeding fly. We did not calculate the achromatic contrast between loci of *E. tenax* because there is little behavioural evidence of the relative importance of achromatic vision compared with chromatic vision (but see An *et al.*, 2018).

Using the spectra from each part of the flower, quantum catches for both the bee and fly models were calculated with the following formula:


$$P=R\;\int_{300}^{700}\;S_{i}(\lambda )I(\lambda )D(\lambda )d\lambda$$

where $S_{i}$ is the sensitivity of each photoreceptor, I(λ) is the spectral reflectance and D(λ) is the daylight illuminant. Quantum catches were hyperbolically transformed using:


$$E\;=\;\frac{P}{P+1}$$

and plotted into hexagonal space using:


$$\chi =\sin60^{\circ }\;\;\times \;(E_{G}-E_{\mathrm{UV}})$$

$$\gamma =E_{B}-\frac{E_{G}-E_{\mathrm{UV}}}{2}$$

to find the *x* and *y* coordinates. The formulas:


$$\chi =E_{\mathrm{UV}}-E_{B}$$

$$\gamma =E_{V}-E_{G}$$

were used to find the coordinates of the relative quantum catches in categorical fly space. All visual modelling used D65 daylight illuminant and green foliage background.

We used a MANOVA on the Cartesian coordinates within the colour vision models to test for statistical differences between the putative ecotypes for each colour trait. This approach accounts for the multivariate nature of the data (Maia and White, 2018). We used the *mvpaircomp* function with a Bonferroni correction from the ‘*biotools*’ package (da Silva *et al.*, 2017) for pairwise comparisons between the putative ecotypes for each colour trait. In cases where there were statistical differences between the putative ecotypes within bee and fly colour vision, we then calculated the Euclidean distances to test whether there were relative perceptual differences between the putative ecotypes (Maia and White, 2018). Empirical means and bootstrapped confidence intervals of colour distances between putative ecotypes were generated using the *bootcoldist* function (Maia and White, 2018). We considered larger distances to be more perceptible than smaller distances. All spectral processing, visualization and colour vision modelling were completed using the R package ‘*pavo*’ (Maia *et al.*, 2019)

### Do putative ecotypes of *G. carneus* occupy distinct biotic and abiotic niches?

#### Point locality sampling from iNaturalist

We extracted flowering and point locality data for all putative *G. carneus* ecotypes from iNaturalist observations, which had >1500 observations as of September 2024. The flowering data were used to test for differences in the phenological niche between putative ecotypes, and the point localities were used to extract abiotic data from environmental layers that test for differences in the realized and fundamental abiotic niche between putative ecotypes. The observations include images of the flowers, allowing for reliable identification of the putative ecotypes, and the associated locality and accuracy data provide reliable occurrence data across its geographical range. Additionally, *G. carneus* is documented yearly on iNaturalist, allowing for multi-year observation dates that can be used to capture temporal variation in flowering times between years and localities. We classified iNaturalist observations into the seven putative ecotypes based on their floral morphology and location. Furthermore, we excluded: (1) any observations that did not have clear photographs of the flowers; (2) any observations of closely related taxa that had been misidentified as *G. carneus*; (3) any observations that did not fit the descriptions of previously identified putative ecotypes or were likely to be hybrids; (4) any observations that occurred outside of the natural range of the species within the CFR (e.g. Australia); and (5) any observations that were not assigned research grade status.

#### Do putative ecotypes of *G. carneus* occupy distinct abiotic niches?

To test whether the putative ecotypes of *G. carneus* occupy distinct abiotic niches, we modelled the predicted distribution of each putative ecotype using MaxEnt (Phillips *et al.*, 2006; Elith *et al.*, 2011). All iNaturalist observations were filtered to include coordinates with an open geoprivacy and an accuracy <100 m to ensure that the niches of each putative ecotype were accurately represented at fine spatial scales. In addition to iNaturalist data, we provided point localities for putative ecotypes that were not well documented, from observations in the field (K. Khoury, unpublished data). Overall, we documented 209 *albidus*, 33 *blandus*, 67 *callistus*, 34 *high-altitude*, 37 *langeberg*, 379 *macowanianus* and 27 *prismatosiphon* point localities to be used in subsequent analyses. We mined 19 bioclimatic layers and an elevation layer at the 30 arc second (∼1 km) resolution from WorldClim (https://www.worldclim.org/data/worldclim21.html), and seven soil layers from Cramer *et al.* (2019). The soil layers from Cramer *et al.* (2019) were modelled specifically in the CFR and allow for more reliable predictions of vegetation type in the CFR than the comparable SoilGrids layers. All stacking, resampling and processing of abiotic layers were done using the ‘*raster*’ package (Hijmans, 2024). The *res* function was used to ensure that resolution of all WorldClim and soil variables matched. All abiotic layers were resampled to match the distribution of the soil layers using the *resample* function. The resampled abiotic layers covered the entire native range of *G. carneus*. The collinearity of all abiotic variables was tested using a Pearson correlation approach, and any abiotic variables with a correlation coefficient >|0.69| were excluded from further analysis. In total, we were left with nine biologically relevant, uncorrelated abiotic variables that were used in the final models. These abiotic variables included isothermality (as a percentage), annual range in temperature (in degrees Celsius), annual precipitation (in millimetres), precipitation seasonality, electrical conductivity (in millisiemens per metre), total nitrogen (as a percentage), extractable phosphorus (in milligrams per kilogram), extractable sodium (in centimoles per kilogram) and elevation (in metres above sea level) (for descriptions of all layers, see Supplementary Data Table S4). We constructed maximum entropy species distribution models for each putative ecotype using the *maxent* function in the package ‘*dismo*’ (Hijmans *et al.*, 2023). The models were implemented using 75 % of the occurrence data for model training and 25 % for model testing, with 10 000 background points (Elith *et al.*, 2011). The *evaluate* function was used to calculate area under the curve (AUC) scores to assess model performance. The AUC scores range from zero to one, with scores closer to one indicating better model performance. The equal sensitivity and specificity threshold was used to create binary maps from the probability predictions of the MaxEnt output using the *threshold* and *reclassify* functions. Values above the threshold were counted as present, whereas values below were counted as absent. A jackknife test was used to determine the most important environmental variable in each model.

We extracted abiotic layers for each point locality to test whether the putative ecotypes occupied distinct realized abiotic niches. We used a PCA to test for niche differentiation, which included the uncorrelated variables used in the niche modelling. We used a permutation MANOVA to test whether the putative ecotypes cluster separately based on their abiotic niche, then a pairwise permutation MANOVA with a Bonferroni correction for pairwise comparisons between the putative ecotypes. The PCA, permutation MANOVA and pairwise permutation MANOVA were implemented using the same procedure described for the morphological analysis.

#### Do putative ecotypes of *G. carneus* flower at different times?

iNaturalist observations were used to document differences in flowering times across all putative ecotypes. Given that iNaturalist records have an observation date, we documented the total number of observations of flowering plants per day of the year as a proxy for the flowering times of each putative ecotype. We pooled observation data across years and associated each observation date with a day of the year (from 1 to 365), which provided a multi-year data set showing the time of year when each putative ecotype was commonly documented, and therefore flowering. In total, we used 307 *albidus*, 59 *blandus*, 129 *callistus*, 51 *high-altitude*, 38 *langeberg*, 612 *macowanianus* and 33 *prismatosiphon* observations from iNaturalist in the temporal analysis.

Given that flowering data are seasonal, best represented as circular data, we used circular statistics to test for differences in flowering times between the *G. carneus* putative ecotypes (Pewsey *et al.*, 2013). To facilitate the analysis, each day of the year (from 1 to 365) was converted into radians, the standard unit of angular measurement. The number of observations against each radian was then used in the analysis. A Mardia–Watson–Wheeler test, a non-parametric test of the homogeneity of two or more samples of circular data, was used to test for significant differences in the flowering times between putative ecotypes. We used a series of Mardia–Watson–Wheeler tests to conduct pairwise comparisons between the flowering times of putative ecotypes. A Bonferroni correction was applied to the resulting *P*-values to reduce the likelihood of type I errors. All analysis was conducted using the *watson.wheeler.test* function in the ‘*circular*’ package (Agostinelli and Lund, 2024).

#### Do putative ecotypes of *G. carneus* occupy distinct pollination niches?

Pollinator observations were conducted across 16 sites of *G. carneus* in 2020, 2022 and 2023 to identify the main pollinators of each putative ecotype. A total of 723 flowers were observed over >69 h (see breakdown of observations per site in Supplementary Data Table S5). Observations were conducted between 08:30 and 14:30 h on days >20 °C. At each site, a maximum of two observers recorded all pollinator visits, the total observed flowers and the total observation time. All visits recorded on focal plants within the observation time were used to calculate visitation rates (as visits per flower per hour) of pollinators at each population. In addition to recording visits, pollinators were caught using an insect net and euthanized using ethyl acetate fumes. These insects were used for species-level identification, the measurements of pollinator functional traits and counting conspecific pollen loads. On each insect, we measured extended proboscis length and thorax depth, because they are likely to be correlated with floral tube length and flower gape. The head, thorax and abdomen of each pollinator were swabbed for pollen using Fuchsin gel, in which the number of pollen grains was counted using a compound microscope and identified as either *G. carneus* or heterospecific pollen, which was determined from pollen controls taken from each population. Pollen loads of >1000 grains on each part of the pollinator were not counted any further. Insects recorded visiting and making contact with the reproductive parts of the flowers or found with *G. carneus* pollen on the head, thorax or abdomen were considered to be legitimate pollinators and used in the subsequent analyses. Once pollinators were identified, they were classified into functional pollinator groups based on similarities in body size, proboscis length and foraging behaviours. The functional pollinator groups identified were solitary bees, carpenter bees, long-tongued flies (LTFs), medium-tongued flies (MTFs), honey bees (*Apis mellifera*) and lycaenid butterflies, which were used for all subsequent analyses.

A series of networks were used to associate the main functional pollinators for each population and putative ecotype. We calculated the visitation rate, mean pollen loads and pollinator importance (visitation rate × pollen loads) per functional pollinator for each site (for sample sizes, see Supplementary Data Table S5). The visitation rates, pollen loads and pollinator importance values were then visualized using seperate networks using the *plotweb* function. For all three networks, H2′ was used to assess the overall level of specialization for the entire network. The H2′ values range between zero (indicating no specialization) and one (indicating complete specialiszation) (Bluthgen *et al.*, 2006). We used a modularity test on the pollinator importance network to identify different pollinator niches for the *G. carneus* populations. The modularity test was conducted using the *computeModules* and *plotModuleWeb* functions (Beckett, 2016). All these analyses used the ‘*bipartite*’ package (Dormann *et al.*, 2008).

We also tested whether there were relationships between floral and pollinator morphology using ordinary least squares regressions, specifically between tube length and proboscis length, and between flower gape and thorax depth. As many sites had more than one functional pollinator, pollinator importance values were used to calculate a ‘weighted’ proboscis length and thorax depth for each site. In the models, the focal floral measurement (either tube length or flower gape) was set as the response, and the pollinator morphology (weighted proboscis length or thorax depth) was set as the explanatory variable. All models were implemented using the *lm* function in the ‘*stats*’ package (R Core Team, 2021).

### Are differences in the ecological niches of the putative *G. carneus* ecotypes resulting in premating reproductive isolation?

#### Calculation of premating barriers

Given that all the barriers documented affect the co-occurrence of the putative ecotypes, we used the first of Sobel and Chen (2014)'s equations to calculate ecogeographic, phenological and pollinator-mediated isolation.

The equation:


$$\mathrm{RI}=1-\left(\frac{S}{S+U}\right)$$

where *S* is the shared and *U* is the unshared portion of occurrence is used to calculate reproductive isolation (RI) for prezygotic barriers that affect co-occurrence. The resulting reproductive isolation values range from zero to one, where a value of zero represents complete overlap and absent reproductive isolation, and one represents no overlap and complete reproductive isolation.

For ecogeographic isolation, we used the binary niche model predictions of each putative ecotype to estimate the shared and unshared predicted niche occupancy for each pair of putative ecotypes. Areas where both putative ecotypes were present were counted as the ‘shared’ area, whereas areas where only one putative ecotype was present were counted as ‘unshared’. When measuring phenological isolation, we were unable to take into account the relative abundances of each flowering putative ecotype as suggested by Sobel and Chen (2014) owing to differences in sampling efforts on iNaturalist. Given that putative ecotypes such as *macowanianus*, *albidus*, *blandus* and *callistus* largely occur in residential areas and on easily accessible hiking routes, they are very well documented. Other putative ecotypes, such as *high-altitude*, *langeberg* and *prismatosiphon*, largely occur in remote or inaccessible areas, resulting in fewer occurrence points, making it difficult to estimate the number of individuals that are flowering in any area. Therefore, instead of using the relative abundance of each flowering putative ecotype, we documented binary flowering occurrences on each day of the year to calculate phenological isolation. We used the number of shared and unshared functional pollinators to calculate pollinator-mediated isolation. All legitimate functional pollinators (i.e. insects recorded visiting a putative ecotype and making contact with the reproductive parts of the flower, or found with *G. carneus* pollen on their head, thorax or abdomen) were used to calculate pollinator-mediated isolation. Given that *blandus* lacked any pollinator data, pollinator-mediated isolation was not calculated for any gene flow direction including that putative ecotype.

Total reproductive isolation was calculated using equation 4E from Sobel and Chen (2014). The equation calculates total reproductive isolation sequentially from prezygotic barriers that affect co-occurrence to prezygotic barriers not affecting co-occurrence and, finally, post-zygotic barriers. Given that only premating barriers have been included in the calculation, the resulting value represents total premating reproductive isolation. Total premating isolation for gene flow directions involving *blandus* included only ecogeographic and phenological isolation. Total premating isolation for all other gene flow directions was calculated using ecogeographic, phenological and pollinator-mediated isolation. For each premating barrier, we calculated 95 % confidence intervals from 10 000 bootstrapped samples of our raw data sets (Ostevik *et al.*, 2016). We also resampled each of the premating barrier distributions 10 000 times to calculate the 95 % confidence intervals for total premating reproductive isolation (Ostevik *et al.*, 2016).

## RESULTS

### Are putative ecotypes of *G. carneus* morphologically distinct?

#### Morphological differences between ecotypes of *G. carneus*

**Fig. 2.**
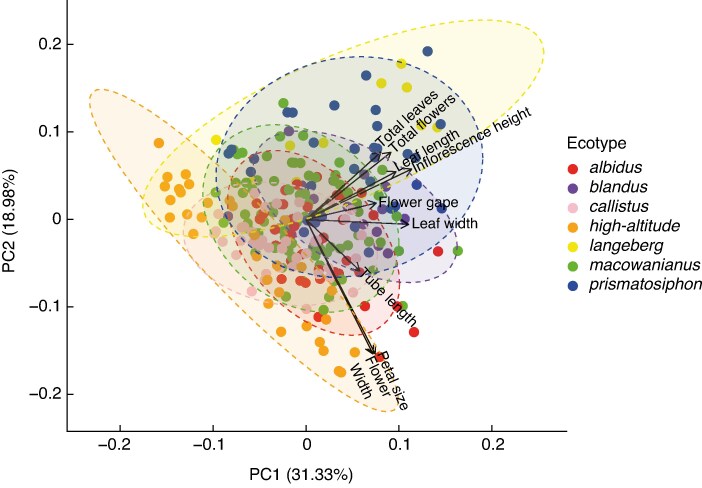
Principal component analysis (PCA) of floral and vegetative traits of *Gladiolus carneus* ecotypes. The PCA includes ellipses showing the 95 % confidence intervals and a biplot of trait loadings. The variables included in the PCA are as follows: tube length, flower gape, flower width, petal size, inflorescence height, leaf length, leaf width, total flowers and total leaves.

The permutation MANOVAs of all morphological traits (*F* = 14.36, d.f. = 6, *P* = 0.001; Fig. 2), floral traits (*F* = 9.38, d.f. = 6, *P* = 0.001; Supplementary Data Fig. S3A) and vegetative traits (*F* = 17.29, d.f. = 6, *P* = 0.001; Supplementary Data Fig. S3B) showed that the putative ecotypes clustered separately, providing morphological evidence for ecotype status in the species complex. The pairwise MANOVA on all morphological traits found that all pairwise comparisons for the ecotypes were significant (*P* < 0.05). The pairwise comparisons on the floral traits showed that all pairings were significant (*P* < 0.05), except for *callistus* and *high-altitude* (*P* = 1.00). The pairwise comparisons between ecotypes based on their vegetative traits showed that all pairings were significant (*P* < 0.05), except between *albidus* and *blandus* (*P* = 0.13) and between *macowanianus* and *callistus* (*P* = 0.19).

We found that both ecotype and site nested within ecotype were significant predictors of tube length (*P* < 0.0001 and *P* < 0.0001, respectively; Supplementary Data Table S6; Fig. S4A), flower gape (*P* < 0.0001 and *P* < 0.0001, respectively; Supplementary Data Table S6; Fig. S4B), petal size (*P* < 0.0001 and *P* < 0.0001, respectively; Supplementary Data Table S6; Fig. S4C), inflorescence height (*P* < 0.0001 and *P* < 0.0001, respectively; Supplementary Data Table S6; Fig. S4D), the leaf length (*P* < 0.0001 and *P* < 0.0001, respectively; Supplementary Data Table S6; Fig. S4E) and leaf width (*P* < 0.0001 and *P* < 0.0001, respectively; Supplementary Data Table S6; Fig. S4F). Furthermore, pairwise comparisons showed that there were significant differences between the morphological traits of the ecotypes (Supplementary Data Table S7).

#### Colour differences between putative ecotypes of *G. carneus*

**Fig. 3.**
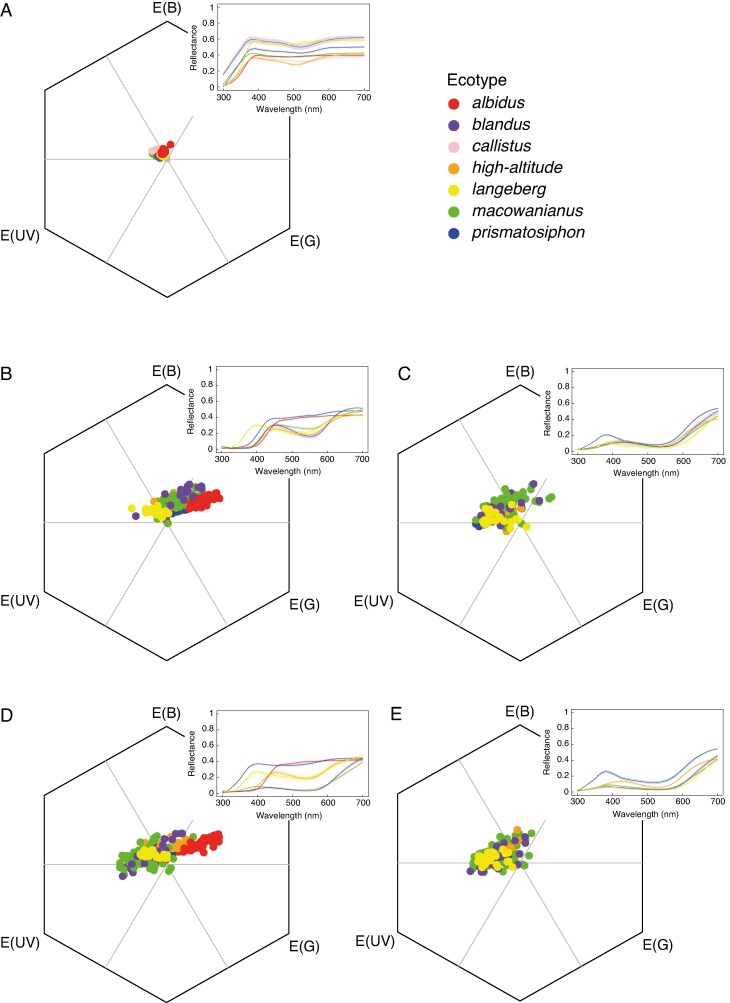
Spectra of *Gladiolus carneus* ecotypes’ tepal (A), centre of the median tepal (B), guide of median tepal (C), centre of lateral tepal (D) and guide of lateral tepal (E) plotted in bee colour vision with *Apis mellifera* spectral sensitivities. The top right of each colour vision model shows the associated spectral reflectance curves for each *G. carneus* ecotype. The mean spectral reflectance curve is depicted by the dark line in the centre, with lighter shading representing the s.e.

**Fig. 4.**
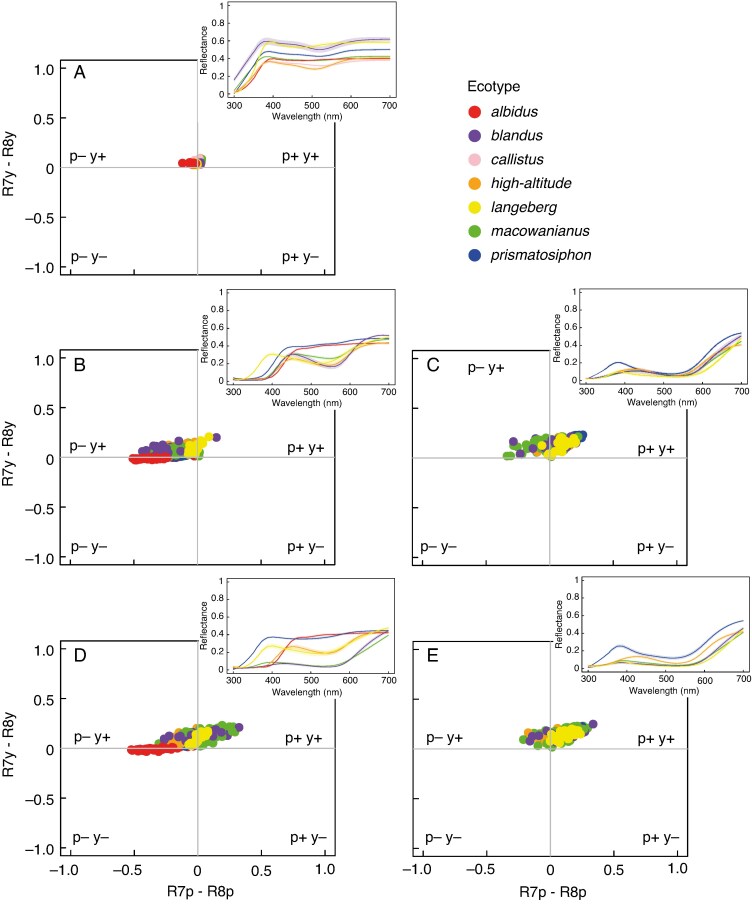
Spectra of *Gladiolus carneus* ecotypes’ tepal (A), centre of the median tepal (B), guide of median tepal (C), centre of lateral tepal (D) and guide of lateral tepal (E) plotted in fly colour vision with *Eristalis tenax* spectral sensitivities. The top right of each colour vision model shows the associated spectral reflectance curves for each *G. carneus* ecotype. The mean spectral reflectance curve is depicted by the dark line in the centre, with lighter shading representing the s.e.

There were statistical differences between the ecotypes for all colour traits modelled in bee and fly colour vision. Specifically, there were significant differences between the ecotypes’ tepals (bee: *Approximate F* = 39.73, d.f. = 6, *P* < 0.0001; fly: *Approximate F* = 38.84, d.f. = 6, *P* < 0.0001; Figs 3A and 4A), centre of the median tepals (bee: *Approximate F* = 45.21, d.f. = 5, *P* < 0.0001; fly: *Approximate F* = 39.74, d.f. = 5, *P* < 0.0001; Figs 3B and 4B), guide of the median tepals (bee: *Approximate F* = 8.57, d.f. = 4, *P* < 0.0001; fly: *Approximate F* = 7.68, d.f. = 4, *P* < 0.0001; Figs 3C and 4C), centre of the lateral tepals (bee: *Approximate F* = 43.78, d.f. = 5, *P* < 0.0001; fly: *Approximate F* = 51.14, d.f. = 5, *P* < 0.0001; Figs 3D and 4D) and guide of the lateral tepals (bee: *Approximate F* = 4.54, d.f. = 4, *P* < 0.0001; fly: *Approximate F* = 3.58, d.f. = 4, *P* = 0.0005; Figs 3E and 4E). Pairwise comparisons showed there were significant differences between the ecotypes for all the colour traits within bee and fly colour vision (see pairwise comparisons in Supplementary Data Table S8). Given that there were statistical differences between the ecotypes for all colour traits, it was necessary to test whether those differences are perceptible within bee and fly colour vision.

The Euclidean distances between the tepals of ecotypes were relatively small in both bee and fly colour vision (Supplementary Data Figs S5A and S6A), suggesting that bees and flies were unlikely to be able to perceive the differences between the tepals of ecotypes. In contrast, the Euclidean distances between the ecotypes’ centre of the median tepal and between lateral tepals were frequently large (Supplementary Data Figs S5B, D and S6B, D), suggesting that both bees and flies were able to perceive the differences between the ecotypes. The Euclidean distances between the ecotypes’ median guides and between the lateral guides were comparatively less perceptible than between the centre of the guides in both bee and fly colour vision (Supplementary Data Figs S5C, E and S6C, E). These results suggest that most of the variation in the colour contrasts between the ecotypes occurs between the centre of the guides.

The achromatic contrasts between the ecotypes’ tepal and between the centre of the median tepals was relatively imperceptible in bee vision (Supplementary Data Fig. S7A, B). However, many of the achromatic contrasts between the ecotypes’ centre of the lateral tepal, guides of the median and guides of the lateral tepals were relatively large, suggesting that these contrasts might be important in allowing bees to distinguish between these flowers (Supplementary Data Fig. S7C–E).

### Do putative ecotypes of *G. carneus* occupy distinct biotic and abiotic niches?

#### Do putative ecotypes of *G. carneus* occupy distinct abiotic niches?

**Fig. 5.**
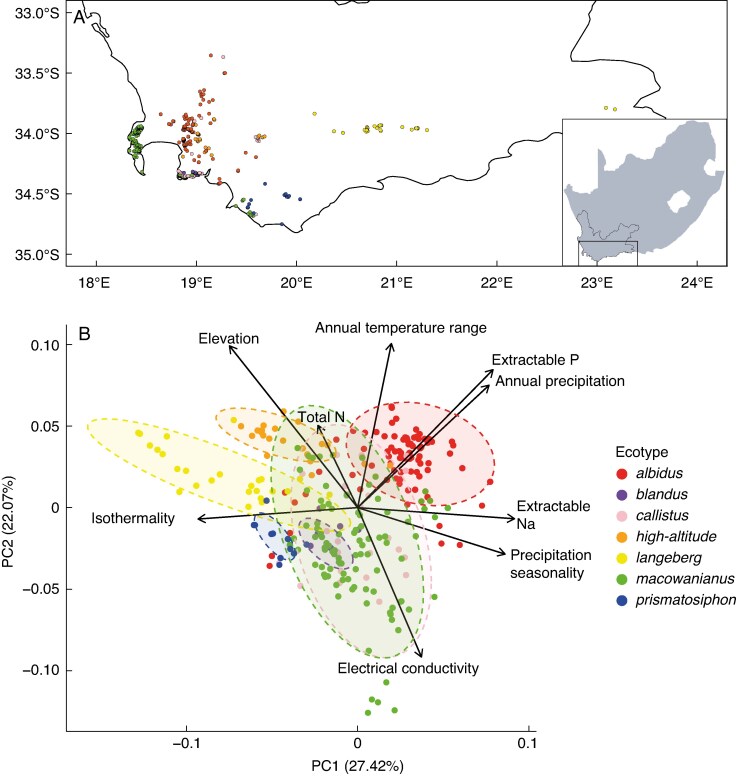
(A) Distribution map of *Gladiolus carneus* ecotypes in the Western Cape of South Africa. All circles on the map show the point localities of all iNaturalist observations used in the environmental niche modes for each *G. carneus* ecotype. (B) Principal component analysis of the abiotic niche of the *G. carneus* ecotypes. Abiotic layers include uncorrelated bioclimatic, elevation and soil variables. The variables include elevation, isothermality, annual temperature range, annual precipitation, precipitation seasonality, electrical conductivity, total nitrogen, extractable phosphorus and extractable sodium.

The MaxEnt models for all ecotypes performed well (AUC > 0.95), providing reliable predicted abiotic niches. All species distribution models closely matched the documented distributions of each ecotype (Fig. 5; Supplementary Data Fig. S8). Specifically, the *albidus* and *callistus* ecotypes had similar distributions around the lowlands of the Boland (Supplementary Data Fig. S8A, C). *Blandus* had the smallest distribution and was largely restricted to the Southern Cape coast between Rooi-Els and Hermanus (Supplementary Data Fig. S8B). The *high-altitude* ecotype was restricted to the Drakenstein, Hottentots-Holland and Riviersonderend mountain ranges (Supplementary Data Fig. S8D). The *langeberg* ecotype was predicted to occur throughout the Langeberg Mountain range (Supplementary Data Fig. S8E). *Macowanianus* had a predicted range covering the peninsula and Southern Cape coast (Supplementary Data Fig. S8F), whereas *prismatosiphon* was largely restricted to the Southern Overberg (Supplementary Data Fig. S8G). The jackknife tests indicated that annual precipitation (in millimetres) was the most important variable in the *albidus*, *callistus* and *high-altitude* models, the annual temperature range (in degrees Celsius) was most important for the *blandus* and *macowaninaus* models, precipitation seasonality was the most important for the *langeberg* model, and extractable phosphorus (in milligrams per kilogram) was the most important for the *prismatosiphon* model. The permutation MANOVA showed that there were significant differences in the realized abiotic niches of the ecotypes (*F* = 100.29, d.f. = 6, *P* = 0.001; Fig. 5B). Furthermore, the pairwise permutation MANOVA indicated that all ecotypes occupied realized abiotic niches that were significantly different from each other (*P* < 0.05).

#### Do putative ecotypes of *G. carneus* flower at different times?

**Fig. 6.**
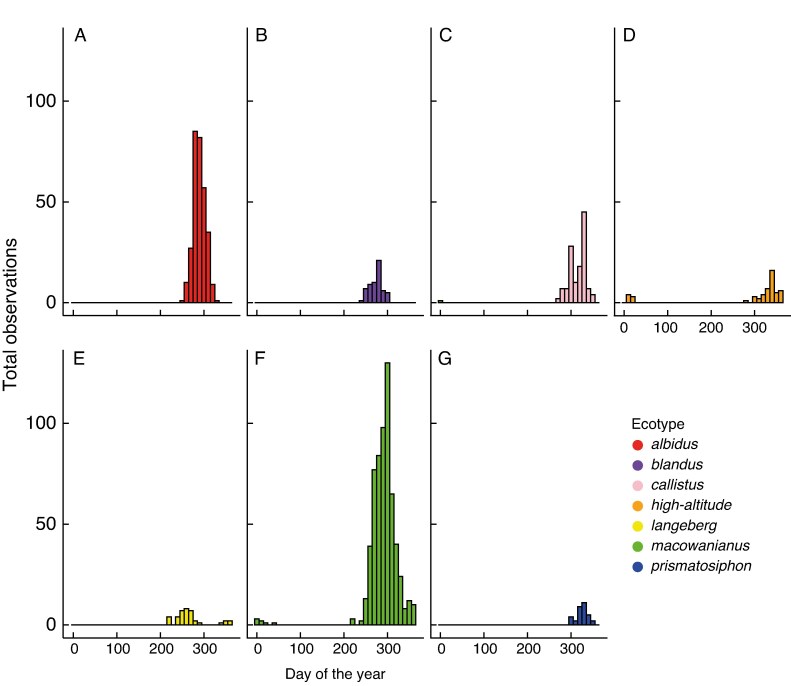
Flowering phenology of *Gladiolus carneus* ecotypes shown by the total iNaturalist observations on each day of the year. *G. carneus* ecotypes are as follows: *albidus* (A), *blandus* (B), *callistus* (C), *high*-*altitude* (D), *langeberg* (E), *macowanianus* (F), and *prismatosiphon* (G).

There were significant differences in the flowering times between *G. carneus* ecotypes (*W* = 389.23, d.f. = 12, *P* < 0.0001; Fig. 6), suggesting that different ecotypes occupy distinct phenological niches. The pairwise comparisons between the flowering times of the ecotypes showed that all pairings were significant (*P* < 0.01) except between *blandus* and *langeberg* (*P* = 0.10) and between *callistus* and *prismatosiphon* (*P* = 1.00).

#### Do putative ecotypes of *G. carneus* occupy distinct pollinator niches?

Across all sampled *G. carneus* populations, a diverse range of pollinators were found, which were subsequently classified into the following functional groups: solitary bees, carpenter bees, long-tongued flies (LTFs), medium-tongued flies (MTFs), honey bees (*Apis mellifera*) and lycaenid butterflies. Solitary bees included *Amegilla spilostoma*, *Amegilla obscuritarsis* (Apidae) and *Anthophora diversipes* (Apidae); carpenter bees included species belonging to the genus *Xylocopa* (Apidae subfamily Xylocopinae); LTFs included *Philoliche rostrata* and *Moegistorhynchus manningi* (Nemestrinidae), which had a proboscis between 28 and 39 mm in length; and MTFs included *Prosoeca nitidula*, *Prosoeca westermanii* (Nemestrinidae) and *Philoliche lateralis* (Tabanidae) which had proboscis lengths between 4 and 16 mm.

**Fig. 7.**
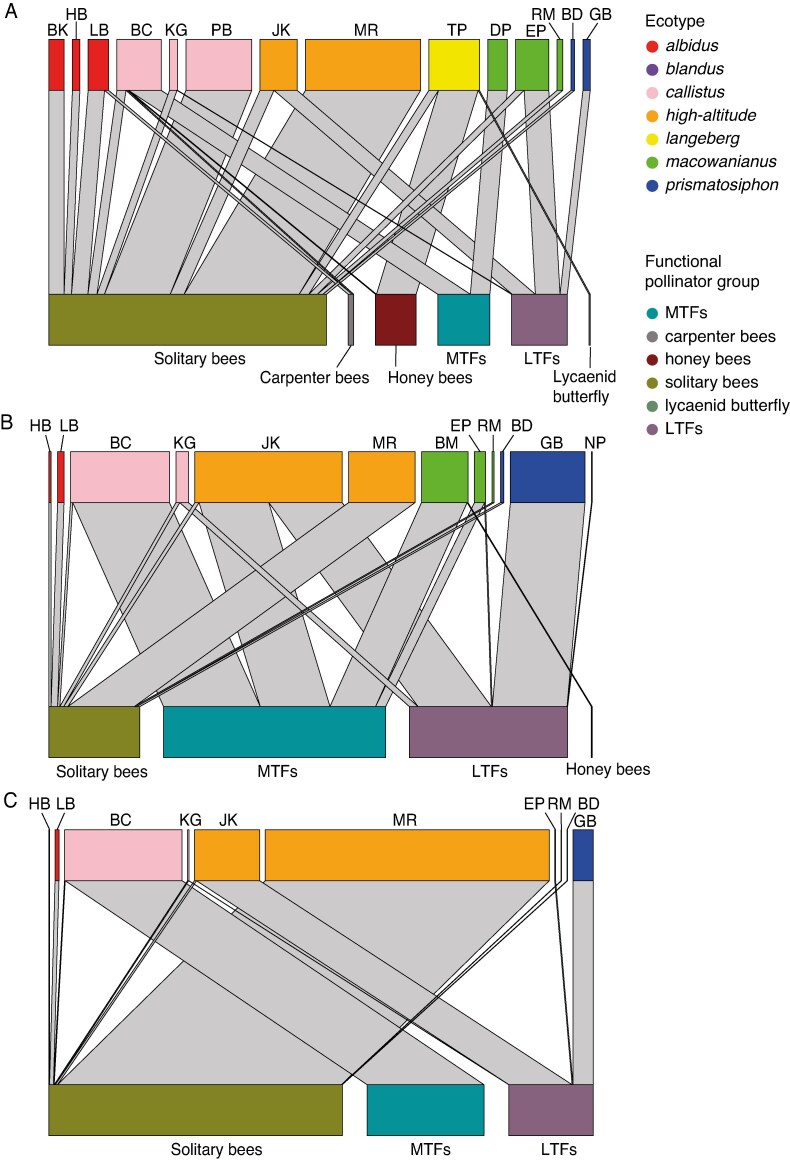
Networks of the visitation rates (A), pollen loads (B) and pollinator importance (C) of the functional pollinator groups: solitary bees, honeybees, carpenter bees, medium-tongued flies (MTFs), long-tongued flies (LTFs), and lycaenid butterflies to different populations of *Gladiolus carneus* ecotypes. Abbreviations above each network refer to distinct populations of *G. carneus* with full site names and coordinates provided in Supplementary Data Table S1.

The networks of visitation rates (*H*2′ = 0.76; Fig. 7A), pollen loads (H2′ = 0.71; Fig. 7B) and pollinator importance (H2′ = 0.99; Fig. 7C) all showed high levels of specialization. In particular, the pollinator importance network showed near complete specialization, with each population having a single highly effective functional pollinator. Across all *G. carneus* populations, there were only three important functional pollinator groups: solitary bees, MTFs and LTFs. The modularity analysis on the pollinator importance network showed that *G. carneus* populations were associated with the same pollinator niches: solitary bees, MTFs and LTFs (Supplementary Data Fig. S9). Although the network and modularity analysis showed high levels of specialization to a specific functional pollinator at the population level, at the ecotypic level the most important functional pollinator was not consistent. Across populations, *callistus*, *high-altitude* and *macowanianus* were all pollinated by MTFs, LTFs and solitary bees; *prismatosiphon* was pollinated by LTFs and solitary bees; *langeberg* was pollinated by solitary bees, honey bees and lycaenid butterflies; and *albidus* was pollinated by solitary bees and carpenter bees.

*Gladiolus carneus* populations show high levels of specialization towards a single functional pollinator, and we also found a significant positive relationship between the tube length and weighted proboscis length (*R*^2^ = 0.46, *F* = 8.622, d.f. = 8, *P* = 0.02; Supplementary Data Fig. S10A); however, there was no significant relationship between flower gape and weighted thorax depth (*R*^2^ = 0.01, *F* = 1.10, d.f. = 8, *P* = 0.33; Supplementary Data Fig. S10B).

### Are differences in the ecological niches of the putative ecotypes of *G. carneus* resulting in premating reproductive isolation?

**Fig. 8.**
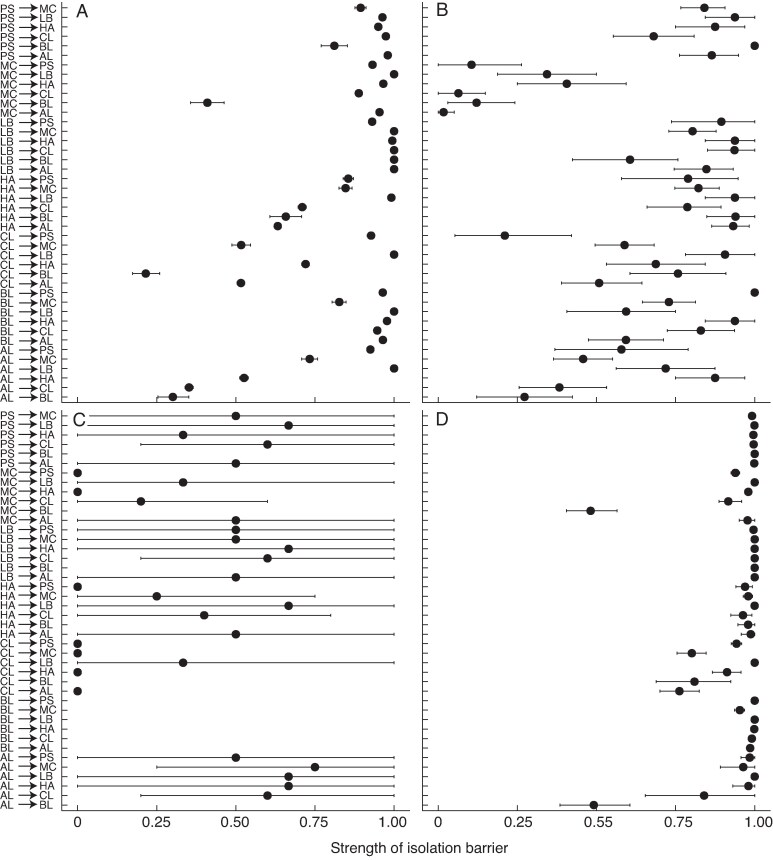
Ecogeographic isolation (A), phenological isolation (B), pollinator-mediated isolation (C) and total premating isolation (D) for all gene flow directions between the *Gladiolus carneus* ecotypes. Each figure includes all gene flow directions between *albidus* (AL), *blandus* (BL), *callistus* (CL), *high-altitude* (HA), *langeberg* (LB), *macowanianus* (MC) and *prismatosiphon* (PS), with arrows indicating directionality. Each point indicates the estimate of reproductive isolation with 95 % confidence intervals based on 10 000 bootstrapped replicates. There are no estimates of pollinator-mediated isolation for gene flow directions including *blandus* owing to a lack of pollinator data. Total premating isolation has been estimated using ecogeographic and phenological isolation for gene flow directions including *blandus*. Premating isolation has been calculated using ecogeographic, phenological and pollinator-mediated isolation for all other gene flow directions.

Ecogeographic isolation was the strongest gene flow barrier across the *G. carneus* species complex (RI_ecogeo_ = 0.83; Fig. 8A). There was weak ecogeographic isolation from *albidus* to *callistus* and the *high-altitude* ecotype (Ecogeographic: RI_al→cl_ = 0.35, RI_al→ha_ = 0.52) and from *callistus* to *albidus* and *macowanianus* (Ecogeographic: RI_cl→al_ = 0.52, RI_cl→mc_ = 0.52). There was also weak ecogeographic isolation from *albidus*, *callistus* and *macowanianus* to *blandus* (Ecogeographic: RI_al→bl_ = 0.30, RI_cl→bl_ = 0.21, RI_mc→bl_ = 0.41). The small range of *blandus* is largely encased within the larger ranges of *albidus*, *callistus* and *macowanianus*, resulting in asymmetric ecogeographic isolation. All other gene flow directions between the ecotypes were >0.6, indicating strong ecogeographic isolation, with many gene flow directions being near complete (Fig. 8A).

Phenological isolation was a relatively strong gene flow barrier across the species complex (RI_phenology_ = 0.67; Fig. 8B). There was weak phenological isolation from *albidus* to *callistus* (Phenology: RI_al→cl_ = 0.27) and from *callistus* to *prismatosiphon* (Phenology: RI_cl→pr_ = 0.21) owing to overlapping flowering times. There was also weak phenological isolation from *macowanianus* to *albidus*, *blandus*, *callistus* and *prismatosiphon* (Phenology: RI_mc→al_ = 0.02, RI_mc→bl_ = 0.12, RI_mc→cl_ = 0.06, RI_mc→pr_ = 0.11). *Macowanianus* has the longest flowering time (stretching from September to early January), resulting in a large overlap in the flowering times of other ecotypes. However, other ecotypes have flowering times that are largely encased within the longer flowering time of *macowanianus*. This overlap occurs for only a short period in the total flowering time of *macowanianus*. This provides a larger opportunity for gene flow from *macowanianus* to other ecotypes than from other ecotypes back to *macowanianus*, resulting in highly asymmetric gene flow.

Pollinator-mediated isolation was a relatively weak gene flow barrier between the ecotypes (RI_pollinator_ = 0.39; Fig. 8C). Given that the sample of shared and unshared pollinators for each ecotype was small, the 95 % confidence intervals for pollinator-mediated isolation were very large, indicating a high degree of uncertainty. Pollinator-mediated isolation was weakest from *callistus* to *albidus*, *high-altitude*, *macowanianus* and *prismatosiphon* (Pollinator: RI_cl→al_ = 0.00, RI_cl→ha_ = 0.00, RI_cl→mc_ = 0.00, RI_cl→pr_ = 0.00); from *high-altitude* to *prismatosiphon* and *macowanianus* (Pollinator: RI_ha→pr_ = 0.00, RI_ha→mc_ = 0.25); and from *macowanianus* to *prismatosiphon*, *high-altitude* and *callistus* (Pollinator: RI_mc→pr_ = 0.20, RI_mc→ha_ = 0.00, RI_mc→cl_ = 0.00). *Callistus*, *macowanianus* and *high-altitude* ecotypes have broad pollinator niches, resulting in more opportunities for gene flow to other ecotypes.

The combined effects of ecogeographic, phenological and pollinator-mediated isolation has resulted in near complete premating isolation across the species complex (RI = 0.95; Fig. 8D). The only weak gene flow direction for total premating reproductive isolation was from *macowanianus* and *albidus* to *blandus* (RI_mc→bl_ = 0.48, RI_al→bl_ = 0.49).

## DISCUSSION

We provided evidence that morphologically distinct ecotypes of *G. carneus* occupy distinct realized and fundamental abiotic niches, resulting in strong ecogeographic isolation across the species complex. The ecotypes additionally had different flowering times that caused moderate phenological isolation. Although individual populations of *G. carneus* were pollinated by a single, highly effective functional pollinator, at the ecotypic level, plants had multiple highly effective functional pollinators, resulting in relatively weak pollinator-mediated isolation. The strength of these premating barriers and, in particular, ecogeographic isolation, caused near complete reproductive isolation between the ecotypes. These results suggested that niche differentiation, and particularly abiotic factors, might be playing a role in driving incipient speciation within the species complex.

### Are *G. carneus* ecotypes morphologically distinct?

All ecotypes showed evidence of being morphologically distinct in both floral and vegetative traits, lending support to the ecotypes initially included by Delpierre and du Plessis (1974) and for the two previously undescribed ecotypes (*langeberg* and the *high-altitude*). The ecotypes also differed significantly in their colour traits. *Albidus* had only a large central ultraviolet-absorbent nectar guide (Supplementary Data Figs S1E and S2C, E), and *callistus* had no nectar guides and instead had a dark purple gullet (Supplementary Data Figs S1F and S2B). All other ecotypes had distinctly coloured centres and guides on their lower tepals (Supplementary Data Fig. S2C–F). The bee and fly colour vision models indicated that most of the perceptible differences between the ecotypes were in the centres of the median and lateral tepals. In particular, *langeberg* and *albidus* had distinct centres on their median tepals, whereas *albidus*, *high-altitude* and *prismatosiphon* had distinct centres on their lateral tepals. These divergent colour properties are likely to be associated with adaptations to distinct functional pollinators (Demarche *et al.*, 2015). In particular, the ultraviolet-absorbent centres of *albidus* are likely to have evolved to attract their pollinators, solitary bees (Chittka, 1992; Demarche *et al.*, 2015; de Camargo *et al.*, 2019; Finnell and Koski, 2021; Newman *et al.*, 2022). The purple and red nectar guides found on all other ecotypes, except for *callistus*, are consistent with nectar guides documented in long-proboscid nemestrinid and *Philoliche* fly systems and might represent a unique adaptation to the long-proboscid fly pollination syndrome (Goldblatt *et al.*, 2001; Newman *et al.*, 2014). However, given that the ecotypes were not associated with a single highly effective functional pollinator, direct associations between nectar guide properties and functional pollinators should be assessed at the population level with reference to the most important pollinator (Moir *et al.*, 2025).

### Abiotic niche differentiation causing ecogeographic isolation

*Gladiolus carneus* ecotypes occupied distinct realized and fundamental niches (Fig. 5; Figure S8), leading to strong ecogeographic isolation across the species complex. There were a number of cases of asymmetric ecogeographic isolation, which occur when there are unequal opportunities for directional gene flow between taxa (Christie *et al.*, 2022), often caused by one taxon having a range largely encased within the other. These cases were caused by: (1) the large *albidus* range overlapping with the *callistus* and *high-altitude* ecotypes; and (2) the small *blandus* range being largely encased within the ranges of *albidus*, *callistus* and *macowaniaus*. Across plant taxa, ecogeographic isolation is relatively symmetrical; however, individual cases can vary substantially, which largely corroborates the results found in the *G. carneus* complex (Christie *et al.*, 2022). These results, along with the differences in vegetative morphology documented between the ecotypes (Supplementary Data Fig. S3B), suggest that abiotic niche differentiation plays a potential role in the diversification of the species complex. The importance of abiotic niche differentiation driving diversification is largely congruent with previous experimental evidence in the CFR (e.g. Latimer *et al.*, 2009; Carlson *et al.*, 2011; Mitchell *et al.*, 2015; Carlson *et al.*, 2016). For example, Carlson *et al.* (2011) found that white *Protea* populations differ in functional traits across environmental gradients, some of which are likely to be maintained by divergent selection imposed by ecological differences. Macroevolutionary evidence supporting abiotic niche differentiation in the CFR is somewhat mixed. van der Niet and Johnson (2009) tested for ecological shifts in 188 Cape sister species and found evidence that habitat, pollinator and fire-frequency shifts were more frequent than soil type shifts, whereas Schnitzler *et al.* (2011) analysed 470 species from the phylogenies of four Cape clades and found that soil type had the highest variability between sister species in three of the clades. Outside of the CFR, abiotic niche differentiation is a major driver of diversification (Grossenbacher *et al.*, 2014; Fernandez-Mazuecos and Glover, 2025), and the resulting gene flow barrier, ecogeographic isolation, has been found to be one of the strongest gene flow barriers between recently diverged taxa (Christie *et al.*, 2022).

### Differences in flowering time causing phenological isolation

There were differences in the flowering times of the *G. carneus* ecotypes, leading to varying strengths of phenological isolation (Fig. 6). In a few cases, phenological isolation was asymmetric, which was largely attributable to *macowanianus* having a long flowering time that overlapped with *albidus*, *blandus*, *callistus* and *prismatosiphon*. These shifts in phenology might also be driving diversification within the species complex. Phenological shifts have been well documented within the CFR, with van der Niet and Johnson (2009) showing that >30 % of 188 species pairs from eight Cape clades had phenological differences. Likewise, Linder (2020) found that 47 % of 112 sister-species pairs in the Restionaceae had non-overlapping flowering times. These findings indicate that phenological shifts seem to be a relatively frequent isolating barrier between taxa in the CFR (Ellis *et al.*, 2014). However, this seems to contradict the results from a global comparison showing that phenological isolation seems to play a lesser role in speciation events between recently diverged taxa (RI_phenology_ = 0.38) (Christie *et al.*, 2022).

### Differing functional pollinators causing pollinator-mediated isolation

Although each population of *G. carneus* had a single highly effective functional pollinator, each ecotype was not associated with a single functional pollinator. This suggests that the ecotypes do not occupy distinct pollination niches and that pollinators are unlikely to be driving the diversification at the ecotypic level. These results are similar to those of Schnitzler *et al.* (2011), who found that in four highly diverse Cape clades, pollinator shifts were relatively infrequent in comparison to shifts in soil type and fire-survival strategy. However, these results contrast with both phylogenetic and experimental evidence showing that pollinator-driven speciation is common, both in the CFR (van der Niet and Johnson, 2009; Johnson, 2010; Anderson *et al.*, 2014; Forest *et al.*, 2014; Newman *et al.*, 2015; Newman and Johnson, 2021) and in the genus *Gladiolus* (Anderson *et al.*, 2010; Valente *et al.*, 2012). Furthermore, Christie *et al.* (2022) found that pollinator-mediated isolation, along with ecogeographic isolation, was one of the strongest reproductive isolation barriers documented across 89 taxa pairs of seed plants. However, given that populations of *G. carneus* had only one highly effective functional pollinator and there were associations between floral and pollinator morphology across sites, pollinators might be driving population-level shifts and causing reproductive isolation between populations rather than between ecotypes. This is further supported by the significant correlations between the weighted proboscis lengths and floral tube lengths across sites (Supplementary Data Fig. S10A), suggesting that there is population-level divergence in some floral traits. Alternatively, the estimates of pollinator-mediated isolation might be inaccurate. Taking into account only the shared and unshared functional pollinators does not account for differential efficiency of functional pollinators to alternative ecotypes (Ostevik *et al.*, 2016; Moir *et al.*, 2025). The strength of pollinator-mediated isolation could be estimated more accurately between ecotypes with sympatric populations (e.g. *macowanianus* and *callistus*), where pollinator transitions (Sobel and Streisfeld, 2015) and pollen flow (Kay, 2006) could be used to estimate behavioural and mechanical isolation.

### Should *G. carneus* ecotypes be treated as separate taxa?

The biological species concept posits that taxa should be treated as separate species when they are reproductively isolated from one another (Coyne and Orr, 2004). Ecotypes of *G. carneus* showed evidence of distinct vegetative and floral morphology (Figs 1 and 2) and are strongly reproductively isolated from one another (RI_total_ = 0.95). The only exception, *blandus*, does not differ from *macowanianus* in morphology and is encased within the range and flowering time of *macowanianus*, potentially leading to weak reproductive isolation. Given that premating reproductive isolation is near complete between the ecotypes, they can probably be treated as separate taxa regardless of the strength of postpollination isolation. In the CFR, there are examples of interfertile orchids being treated as separate taxa (Newman and Johnson, 2025). However, whether these taxa should be treated as separate species depends on whether species are defined as being reproductively isolated or when reproductive isolation is irreversible (Lowry, 2012). If species are defined by whether they are reproductively isolated from one another, premating barriers alone can be used to delineate species. However, divergent selection on traits that mediate reproductive isolation can allow for introgression and result in the collapse of species boundaries in the future (Lowry, 2012). If species are instead defined by whether reproductive isolation is irreversible, separate species would be delineated only when intrinsic postzygotic isolation is complete, which would be tested using a series of controlled crosses. The *G. carneus* ecotypes, apart from *blandus*, meet the criteria of being reproductively isolated from one another, but it remains to be seen whether it is reversible. Although we have provided ecological evidence that quantifies the potential gene flow between these ecotypes, population genetic data would provide insights into the extent of ongoing gene flow and ‘genetic distinctness’ between ecotypes (Westram *et al.*, 2022). This should also be paired with genetic and ecological evidence of whether these ecotypes can exist stably in sympatry (Coyne and Orr, 2004; Mallet and Mullen, 2022).

### Conclusion

Overall, we have provided evidence of seven morphologically distinct ecotypes within the *G. carneus* species complex that occupy contrasting abiotic and biotic niches, resulting in near complete premating reproductive isolation. These results suggest that niche differentiation is likely to have played a key role in driving the diversification both within this species complex and within the CFR. Future research should test for local adaptation using reciprocal translocations and common gardens, which would provide further evidence of ecological divergence. These field studies should be paired with greenhouse experiments that identify the ecological factors that cause the morphological and niche differentiation between the ecotypes. Additionally, postpollination isolation, specifically a series of controlled crosses, should be quantified between these ecotypes to determine both its relative contribution to maintaining reproductive isolation and whether reproductive isolation is reversible. These experimental approaches should be paired with phylogenomic and population genetic data to provide evidence of the evolutionary relationships and gene flow patterns within the species complex. This integrated approach would provide insights into the ongoing divergence within this species complex and the wider CFR.

## Supplementary Material

mcaf172_Supplementary_Data

## Data Availability

All data associated with this manuscript can be found via on DYRAD at doi: 10.5061/dryad.0cfxpnwf5.

## SUPPLEMENTARY DATA

Supplementary data are available at *Annals of Botany* online and consist of the following. Figure S1: morphological measurements of *Gladiolus carneus* ecotypes. Figure S2: spectral reflectance curves for the tepal (A), gullet (B), centre of the median tepal (C), guide of median tepal (D), centre of lateral tepal (E) and guide of lateral tepal (F) for each *Gladiolus carneus* ecotype. Figure S3: principal component analyses (PCA) of floral (A) and vegetative (B) traits of the *Gladiolus carneus* ecotypes. Figure S4: comparisons of the functional traits tube length (A), flower gape (B), petal size (C), inflorescence height (D), leaf length (E) and leaf width (F) between *Gladiolus carneus* ecotypes. Figure S5: Euclidean distances between each ecotype for the tepal (A), centre of the median tepal (B), guide of median tepal (C), centre of lateral tepal (D) and guide of lateral tepal (E) modelled in bee colour vision with *Apis mellifera* spectral sensitivities. Figure S6: Euclidean distances between each ecotype for tepal (A), centre of the median tepal (B), guide of median tepal (C), centre of lateral tepal (D) and guide of lateral tepal (E) modelled in fly colour vision with *Eristalis tenax* spectral sensitivities. Figure S7: achromatic contrasts between each ecotype for the tepal (A), centre of the median tepal (B), guide of median tepal (C), centre of lateral tepal (D) and guide of lateral tepal (E) modelled in bee colour vision with *Apis mellifera* spectral sensitivities. Figure S8: niche model outputs for *albidus* (A), *blandus* (B), *callistus* (C), *high-altitude* (D), *langeberg* (E), *macowanianus* (F) and *prismatosiphon* (G) based on uncorrelated bioclimatic, elevation and soil layers. Figure S9: modularity analysis of pollinator importance showing *Gladiolus carneus* sites associated with each functional pollinator group. Figure S10: correlations between: (A) the weighted proboscis length (in millimetres) of pollinators and *Gladiolus carneus* tube length (mean ± s.e.; in millimetres); and (B) weighted thorax depth (in millimetres) of pollinators and *G. carneus* flower gape (mean ± s.e.; in millimetres) across populations of *G. carneus*. Table S1: coordinates, and elevation (in metres) of all 29 *Gladiolus carneus* sites sampled for this manuscript. Table S2: sample sizes of the morphological trait measurements at each *Gladiolus carneus* study population. Table S3: sample sizes of individuals for which spectral reflectance were collected for each *Gladiolus carneus* study population and ecotype. Table S4: bioclimatic and topography layers mined from Worldclim and the soil layers from Cramer *et al.* (2019) that were used in the abiotic niche analysis. Table S5: mean and sample size of visitation rates and pollen loads for each functional pollinator collected across populations and ecotypes of *Gladiolus carneus*. Table S6: generalized linear model outputs testing for differences in morphological traits between the *Gladiolus carneus* ecotypes. Table S7: pairwise comparisons between the morphological traits of *Gladiolus carneus* ecotypes. Table S8: pairwise comparisons between ecotypes of *Gladiolus carneus* plotted in bee and fly colour vision models.
